# Health-related shame: an affective determinant of health?

**DOI:** 10.1136/medhum-2017-011186

**Published:** 2017-06-08

**Authors:** Luna Dolezal, Barry Lyons

**Affiliations:** 1 University of Exeter, Exeter, UK; 2 Trinity College Dublin, School of Medicine, Dublin 2, Ireland

## Abstract

Despite shame being recognised as a powerful force in the clinical encounter, it is underacknowledged, under-researched and undertheorised in the contexts of health and medicine. In this paper we make two claims. The first is that emotional or affective states, in particular shame, can have a significant impact on health, illness and health-related behaviours. We outline four possible processes through which this might occur: (1) acute shame avoidance behaviour; (2) chronic shame health-related behaviours; (3) stigma and social status threat and (4) biological mechanisms. Second, we postulate that shame's influence is so insidious, pervasive and pernicious, and so critical to clinical and political discourse around health, that it is imperative that its vital role in health, health-related behaviours and illness be recognised and assimilated into medical, social and political consciousness and practice. In essence, we argue that its impact is sufficiently powerful for it to be considered an affective determinant of health, and provide three justifications for this. We conclude with a proposal for a research agenda that aims to extend the state of knowledge of health-related shame.

## Introduction

Although, shame has been identified as a powerful force in the clinical encounter, and the experience of illness,^1^ ^2^ curiously it remains both undertheorised and commonly unacknowledged in the contexts of health and medicine.^3^ As Darby *et al* 4 note, despite shame's ‘frequent occurrence’ within healthcare settings, there is ‘a surprising lack of research’ examining the effect of shame and other negative self-conscious emotions. Commenting on this apparent mismatch of clinical importance and medical disinterest, Davidoff^3^ dubbed shame the ‘elephant in the room’ in healthcare contexts. However, other disciplines (notably psychology, philosophy and the social sciences) have been less reticent in engaging in a more rigorous analysis of the causes and consequences of shame. Emerging literatures in these disciplines highlight that shame is often significant when considering an individual's health status.

Our aim in this paper is twofold. First, we propose to use insights from the aforementioned emerging literatures, and from the empirical work of others, to further the claim that emotional or affective states, in particular shame, can have an impact on health, illness and health-related behaviours. In so doing, we will contend that body or disease-related shame contributes to the burden of illness, and that shame may itself be a direct cause of ill-health. In order to underpin this claim, we will outline a number of processes or mechanisms through which shame may act on the health of individuals. We schematise these as follows: (1) acute shame avoidance behaviour; (2) chronic shame health-related behaviours; (3) stigma and social status threat and (4) biological mechanisms. These headings are simple descriptors and are not intended to convey the impression that they refer to stand-alone entities. Rather, all are substantially interlinked but have been individuated here in order to provide conceptual clarity.

Second, we postulate that there is a plausible case to be made for shame to be considered as a determinant of health. WHO defines health determinants as ‘the range of behavioural, biological, socioeconomic and environmental factors that influence the health status of individuals or populations’.^5^ Among the array of determinants that are identified are the social, physical and economic environment, and a person's individual characteristics. Although emotional traits or behaviours are not mentioned, there seems no good reason to disallow affective states from being regarded as significantly health impacting. In essence, we argue that shame is so pervasive, so corrosive of the self and so potentially detrimental to health, that there is considerable utility in considering it an affective determinant of health. We include an assessment of the implications of our thesis, including those for the individual doctor-patient encounter, and for public health policy in general.

Finally, we outline a number of research pathways that may be pursued to further investigate health-related shame. On a terminological note, within this paper we use ‘affect’ and ‘emotion’ as interchangeable terms. This may be inherently problematic for some theorists who hold that they represent different concepts. However, in agreement with Greco and Stenner,^6^ we do not feel the distinction is inherently helpful or particularly revelatory, in this context.

## Shame

Shame is regarded as being a negative emotion that arises when one is seen and judged by others (whether they are present, possible or imagined) to be flawed in some crucial way, or when some part of one's self is perceived to be inadequate, inappropriate or immoral. It is what is called a self-conscious emotion in that the object of shame is oneself and, furthermore, it involves an awareness of how other people view the self.^7^ ^8^ Shame experiences are varied and multiple in their expression. As a result, ‘shame’ is often used as an umbrella term to indicate a whole ‘family of emotions’ ranging from ‘the mildest twinge of embarrassment to the searing pain of mortification’.^9^ Hence, shame variants include a wide array of negative self-conscious experiences such as embarrassment, humiliation, chagrin, mortification, feelings of defectiveness or low self-worth.^10^ ^11^ The intensity and expression of a shame experience depends on an extensive constellation of factors including one's culture, background, family experiences, personality and the immediate context. In general, shame is distinguished from guilt by a focus on the person rather than the act. Guilt arises where one feels bad about an action or something that one has done, shame is about the person that one is.^9^

It is argued that because shame is linked to one's core identity, it is among the most powerful and significant affective experiences.^9^ In a moment of shame, one feels flawed or inferior, and feels as though others can see this also. As a result, shame is not just linked to threats to one's identity, but, significantly, it is linked to threats to social bonds.^12^ Shame can threaten one's feelings of belonging and acceptance within interpersonal contexts^13^ and socially and politically.^14^ Shame, as a result, is an alienating and isolating experience that is far from trivial, often deeply disturbing and a cause of significant distress. As Gehert Piers notes, ‘behind the feeling of shame stands [the] fear of abandonment, the death by emotional starvation’.^15^ ^16^ Northrop^17^ echoes this claim contending that in cases of shame and stigma, ‘social death and actual death are imminently convergent’. The threat of shame can feel worse than the threat of physical pain or even the risk of death.^9^ ^18^

While shame signals a significant social threat, it also creates a bind for the person experiencing it, as revealing that one is experiencing shame is itself shameful. As a result, shame symptoms provoke a shame spiral or ‘loop’, in which, when shame arises it incites more shame.^12^ Shame, thus, is an iterated emotion; its occurrence leads to an intensification or multiplication of itself.^19^ ^20^ Consequently, shame is an emotion that is usually fastidiously avoided (both individually and collectively) and if that is not possible, it is scrupulously ignored. Individuals go out of their way to avoid shame (or even mention past instances of shame),^21^ even when this avoidance means harming or hurting the self.

Beyond remaining silent or being scrupulously circumvented, shame is often an ‘unidentified’ or ‘hidden’ emotion which does not enter conscious awareness but is nonetheless frequently present.^19^ Although the experience remains available to consciousness, the person experiencing it is not able to, or perhaps simply will not, identify it as shame, and there is an intrinsic connection between shame and the mechanism of denial. In these cases, shame is ‘by-passed’ and other affects, such as anger, guilt, depression, doubt or excessive displays of pride through narcissism, take over.^14^ ^22^ It is well theorised that when shame occurs, or even when it is merely anticipated, powerful ‘scripts’, or ‘basic patterns of behaviour that govern our reactions’ to it, are activated.^9^ While some of these scripts allow the shame experience to manifest, others use denial or bypassing as coping mechanisms. Nathanson^9^ describes four basic shame scripts: shameful withdrawal, masochistic submission, narcissistic avoidance of shame and the rage of wounded pride. Through this schema of reaction patterns, a wide range of behavioural forms emerge that help one cope with the perceived threats to one's social bonds and one's identity that shame experiences, no matter how mild or intense, provoke.

What is common to this diverse collection of responses and reactions is that shame creates a sense of heightened visibility and, as a result, has a tendency to provoke concealment—to hide one's shame and to obscure that of which one is ashamed. At the same time, shame also provokes ‘a cognitive shock’ to use Nathanson's^23^ formulation, that ‘manages to derail cognition [and] higher cortical function’. In short, shame (or even just the threat of shame) induces a panic state where the ‘necessity’ (to hide or conceal) that shame produces overrides rational thought and moral reasoning.^24^ ^25^ In its painful state of exposure and continuous self-reference, a shamed individual often does not have the cognitive resources to act as a responsive and responsible agent, authentically attuned to the needs of itself and its community of others.^26^ Thus, shame is commonly avoided and hidden, even when these measures potentially entail harming the self or others.

## Health-related shame

That affective states impact on health and illness was something well understood by ancient physicians. Both Hippocrates' theory of the four humours, and Galen's concept of the ‘passions’, point to a significant historical appreciation of the inter-relationship between emotions and morbidity and mortality. Indeed, ‘severe emotional reactions’ were regarded as causative of diseases such as ‘stroke, deformed births, madness, asthma, ulcers and even death’.^27^ Although this idea commanded medical thinking for much of early civilisation, it largely fell into abeyance during the enlightenment. As Sternberg observes: ‘the notion that emotions could have something to do with disease came to be viewed by modern science with disdain and mild amusement—magical thinking, certainly not ideas to be welcomed into the domain of the serious scientist’.^27^ On the rare occasions when emotion was directly addressed, ‘it was typically associated with the primitive, the embodied, the female’.^6^

As a result of these tendencies, there is a paucity of modern scientific writing on shame and its effects in the context of healthcare and medicine. However, it has recently been acknowledged that patients often regard their illnesses as personal shortcomings, or as arising from personal inadequacies.^1^ This is not surprising, as many illnesses continue to carry significant stigma^28^ and ‘flaws’ in the physical body can often be construed as a mark of disgrace, disqualifying, as Goffman^29^ puts it, ‘an individual … from full social acceptance’. The experience of health-related stigma is crucially bound up in experiences of shame,^30^ where threats to one's identity and one's social bonds through carrying a stigma mean that, as Goffman^29^ notes, ‘shame becomes a central possibility’. However, health-related shame is not simply about feelings of inadequacy (no matter how undeserved) with respect to the ailing body. Instead, findings across a broad spectrum of social science and biological research demonstrate that shame impacts on health through a variety of inter-related pathways. As stated in the introduction, we have schematised these as follows: (1) acute shame avoidance behaviour; (2) chronic shame health-related behaviour; (3) stigma and social status threat and (4) biological mechanisms.

### Acute shame avoidance behaviour

Acute shame is the experience of an isolated episode of shame (to be distinguished from chronic shame, which will be discussed below). Acute shame can arise unexpectedly, as in cases of embarrassment where in social interaction, one's self-presentation falters, fails or falls short of socially desired modes of comportment, such as a temporary or unexpected loss of control of one's body and bodily functions.^10^ ^31^ However, acute shame can also be anticipated, for instance, when one expects a moment of exposure or anticipates a mishap or transgression. Acute shame episodes are uncomfortable and alter one's interactions with others while immediately diminishing one's sense of social worth (whether fleetingly or permanently). As a result, individuals go to great lengths to avoid or circumvent acute shame and potential instances of shameful exposure, even when this avoidance might mean harming the self or others.

Acute shame is a distinct possibility in clinical contexts as the exposure of one's (perceived) flaws, inadequacies, faults or frailties are at the heart of many clinical encounters.^1^ ^32^ Lazare^1^ notes that patients often experience their bodily afflictions or diseases as personal defects. Becoming ill, ageing, disfigurement, infectious diseases, mental health issues, obesity, incontinence and other ‘failings’, where one's physical or mental self deviates from an imagined ideal norm of health, youth, fitness and (increasingly) attractiveness, can be potent sources of shame.^33^ Furthermore, salient identity markers which may carry shameful stigma (such as one's socioeconomic status, one's sexuality or literacy levels), or certain stigmatised behavioural patterns (eg, smoking, overeating or poor hygiene) may be revealed by the personal exposure an encounter with a healthcare professional usually entails.^34^ ^35^

Empirical research demonstrates that threats of acute shame regarding one's health, physical body, identity, behaviour or social status can have a significant effect on the process of the clinical encounter.^4^ ^34–36^ Harris and Darby report that of a large cohort of patients, half of all respondents recalled one or more interactions with a physician that left them feeling ashamed. Not all of these felt that being shamed had been a bad thing, but the corollary of this was that even those who deemed the experience to have improved their health behaviour were significantly more likely to lie to their physician subsequently.^34^ Commonly, it is reported that shame, or the anticipation of a shame episode, invokes a tendency towards concealment and avoidance in healthcare contexts and as a result shame can act as an invisible barrier to the adequate delivery of healthcare. When individuals feel the threat of shame this can lead to (i) failure to seek treatment; (ii) failure to disclose the full details of one's mental or physical ill-health or one's situation or identity—for example, one's sexuality or literacy status^35^ ^37^—which may result in inadequate or ineffective treatment being prescribed; (iii) failure to complete the course of prescribed treatment or (iv) diagnosis concealment from family and friends.^30^ The anticipation of painful exposure, along with the fear of the judgement that it might incur, can lead individuals to avoid seeking medical attention or from accurately narrating or disclosing symptoms or histories ‘even when they are concerned about serious symptoms’.^36^ Hence, avoiding potential instances of acute shame can feel like a life-saving measure, even when it puts one's health or life at risk.

### Chronic shame health-related behaviours

Chronic shame differs substantially from the acute shame that arises because of a discrete moment of exposure. Instead, chronic shame involves recurring or persistent shame that forms a background of social pain and self-consciousness; it is an ‘affective attunement’ that colours all aspects of one's life.^38^ As Pattison notes: ‘chronic shame [can shape] a whole personality and may last a lifetime’.^14^ The persistent feeling of inferiority and social exclusion that comes with chronic shame means that it can become debilitating or even pathological, affecting one's life chances, one's relationships and, as recent research has demonstrated, one's health outcomes.^22^

Chronic shame can arise through several mechanisms (that may nonetheless co-occur). First, childhood relational trauma which may encompass experiences such as childhood abuse or highly dysfunctional shame-based family dynamics.^22^ Second, minority stigma, where a salient aspect of one's identity—such as gender, health status, disability, race, sexuality, weight, ethnicity—is stigmatised or is seen to deviate from a centrally valued cultural or social norm.^39–43^ Third, chronic shame is a feature of certain psychopathologies such as post-traumatic stress disorder,^44^ body dysmorphic disorder,^45^ social anxiety disorder^22^ and pathological narcissism.^46^ Whatever the root of one's chronic shame, its manifestation is far from trivial. Pattison terms it a ‘toxic unwantedness’ that results from experiencing (or even mistakenly perceiving) persistent rejection by others because one believes one's identity to be flawed or sullied in some way.^14^ The pervasive perception that one is worth less than others, along with the recurring heightened self-consciousness, negative affect and cognitive shock that shame produces, is profoundly dysfunctional and disempowering, not to mention psychologically damaging.^47^ While in the next section we will discuss how chronic shame arising from minority stigma impacts on health outcomes, here we will outline the evidence that suggests that chronic shame, and most significantly strategies or scripts for its avoidance, can directly impact on health and health relevant behaviour.

In order for an individual to cope with chronic shame, it is frequently bypassed for other experiences and powerful shame ‘scripts’ take effect as a protective measure in order to bypass or avoid the direct experience of shame which may itself be too painful or psychologically damaging.^22^ Through engaging in avoidance strategies, it has been demonstrated that chronic shame is directly implicated in a wide range of health relevant behaviours. There is a growing body of work that implicates chronic shame with risk behaviours such as alcoholism,^48^ addiction^22^ ^47^ and eating disorders,^49^ conditions all of which act to ‘numb’ an individual against the pain of shame. Furthermore, avoidance scripts that bypass shame in favour of narcissism or rage responses have been shown to lead to antisocial behaviour such as violence, bullying and sexual assault.^50–52^ At the same time, withdrawal and avoidance scripts mean that chronic shame commonly leads to states such as stress and anxiety^53^ or depression,^54^ where an individual may not even be aware that they are experiencing shame and instead report other emotional troubles or psychopathological symptoms.^14^ ^22^ As such, chronic shame is clearly linked to behaviours and psychological states that have a direct negative impact on health.

Of course, those burdened with chronic shame can experience episodes of acute shame. It seems reasonable to suggest that chronic shame may lead to an increased sensitivity to acute episodes—for example, a woman suffering chronic shame as a consequence of childhood sexual abuse might well experience a routine gynaecological examination as more traumatic or exposing than another woman who does not share a history of abuse.^55^ It does seem possible that chronic shame might expand the range of possible sources of acute shame, or even the depth or magnitude of shame experienced. However, the hypothesis that acute on chronic shame may be qualitatively or quantitatively different to acute shame arising de novo has not been particularly well examined to date, and thus remains a relatively unsupported theoretical consideration.

### Stigma and social status threat

As noted above, chronic shame is frequently associated with minority stigma, where a salient aspect of one's identity—such as gender, health status, disability, race, sexuality, weight or ethnicity—is stigmatised, and as a result one belongs to a marginalised group or community. In this way, chronic shame often has its roots in the cultural politics of inclusion and exclusion where for certain groups of individuals ‘stigmatising shaming’^56^ is experienced as a frequent, if not constant, background to daily life. In marginalising circumstances, shame becomes deeply embedded and chronically reoccurring. The idea is that even when shame is not felt directly, it is permanently anticipated as one's identity (according to the dominant social, cultural or political norms) is spoiled in the first instance.

It has been well-documented through the minority stress literature that ‘individuals from stigmatised social categories’ experience ‘excess stress’ as a result of ‘their social, often a minority, position’.^57^ These stressors are unique (in that they are over and above those experienced by the non-stigmatised population), they are chronic and, furthermore, arise from social and structural forces and circumstances (rather than as a result of an individual's inherent identity, circumstances or behaviour). The stigma processes identified in the minority stress literature, such as internalised negative self-conception, rejection anxiety, hypervigilance in social situations and concealment behaviours,^37^ clearly follow the patterns of shame and shame avoidance. In fact, minority stress is directly correlated to the experience of chronic shame. For example, researchers have found a substantial racial difference in psychosocial stress, demonstrating that African Americans, among other socially and economically marginalised communities, are more likely to suffer from chronic shame and social-evaluative threats as a result of racism and discrimination.^56^ ^58^ Similar links between a stigmatised social status and chronic shame are found when considering sexuality,^41^ socioeconomic status^42^ and body size.^39^

It is now well established in health research literature on socioeconomic inequalities in health that, by almost any measure, those who are lower down the social hierarchy—in other words, those who suffer from regular stigma, discrimination and marginalisation because of minority status—have poorer health and shorter life expectancy.^59^ A host of different causes have been postulated to explain the higher incidences in poorer health behaviours and outcomes, including cultural differences and differences in material environments, where factors such as poor housing and living environments along with limited economic and social resources cause psychological strain and negative physical health consequences.^42^ While the link between marginalisation and poorer health outcomes is certainly related to material factors, such as limited access to healthy affordable food, lack of opportunities for exercise and restricted access to health services, for example, recent research has revealed that psychosocial processes associated with the experience of low self-esteem and psychophysiological stress resulting from chronic shame, marginalisation and stigma are also significant factors in determining health outcomes and well-being.^59^

The minority stress theory, along with Richard Wilkinson's ‘status anxiety’ hypothesis,^60–63^ argue that social inequalities damage individual health. What has been demonstrated empirically is that minority stress and forms of status anxiety lead to health disparities across populations, where stigmatised populations have higher incidents of mental and physical health problems and are more likely to engage in health risk behaviours. There is a clear empirical correlation between status anxiety—where shame is chronically anticipated or experienced—and deleterious behaviours, such as addiction, violence, criminal inclinations and self-harm, which directly affect health and life expectancy.^50^ ^63^ Experiencing social inequality is also associated with higher rates of infant mortality, very low birth weight in infants,^57^ shorter height, AIDS, depression and poor self-reported health.^63^ In short, social marginalisation, or even just the fear or anticipation of social rejection, through chronic shame and stigma is, it seems, itself a cause of poor health.

### Biological mechanisms

In their review of ‘emotions, morbidity and mortality’, Kiecolt-Glaser *et al* 64 read the biological evidence as strongly supporting the idea that ‘negative emotions can intensify a variety of health threats’ through a number of immune and endocrine responses. Experiences of chronic shame, minority stress and status anxiety cause prolonged stress in the body which has a clear effect on many physiological systems, such as the immune and cardiovascular systems.^63^ An increase in what has been termed ‘social-evaluative threat’, or threats to self-esteem or social status, directly correlate with increased anxiety and heightened biological stress responses.^53^ ^63^ The biological response to stress includes the release into the bloodstream of the individual of various hormonal and chemical mediators including the steroid hormone cortisol and immunologically active substances called pro-inflammatory cytokines (PIC). This response is similar to the ‘fight-or-flight’ mechanism, which is an adaptive response that tells our bodies to flee when we are faced with physical danger.^65^ However, chronic or maladaptive elevations of these agents, resulting in immunological or endocrine dysregulation, can be harmful to health.

Empirical research has clearly demonstrated that shame, and other ‘disengagement-related affective states’, where the subject wants to withdraw, hide or avoid social interaction, causes the body to release cortisol^66^ and PIC.^67^ Stressors which include a social-evaluative threat ‘in which others could negatively judge performance … provoked larger…changes than stressors without these particular threats’.^68^ The response of the body to these endogenous chemicals is complex, being both protective and signalling the brain to modify social behaviour.^69^ Stimulating the release of these substances in ‘healthy’ volunteers produces self-reported feelings of depressed mood and isolation (more commonly and in a more pronounced way in women than men).^70^ It has been proposed that these physiological responses, in acute episodes, serve to protect the individual from threats to social bonds, ensuring that group membership—and hence, physical, emotional and social survival—is maintained.^71^ As a result, it is suggested that these responses can serve to (i) help the individual become more sensitive to endangering social experiences, thus leading to better identification and avoidance of such threats in the future,^72^ while (ii) stimulating others who are close to the affected individual to provide support, care and assist in recuperation.^73^ ^74^

However, with chronic shame, these levels can be persistently altered leading to a variety of negative health effects as a result of the physiological strain on the body and its systems because of chronically elevated PIC and cortisol levels. A variety of conditions, such as ‘weight gain, heart disease, hardening of the arteries and decreased immune function’,^56^ ^63^ have been associated with the physiological responses associated with chronic shame. Moreover, recent research has demonstrated a direct correlation between ‘negative characterological self-appraisals associated with shame’ and ‘immune decline’.^67^ For instance, in a study of HIV-positive patients, shame and perceived threats to one's social bonds clearly correlated with disease progression and mortality.^65^

The purpose of this section is not to argue that shame stimulates some unique biological system that results in negative health outcomes. Rather, shame appears to act, as many other chronic stressors do, through a common pathway that is ultimately injurious in many instances. The notion that affective states can impact on health is further reinforced by research on positive emotions. In general, positive emotions have been shown to be associated with ‘greater longevity and reduced morbidity’.^75^ As might be predicted, positive emotions have the opposite effect on inflammatory activity to negative affective states—positive affect appears to induce a reduced inflammatory response (the available evidence indicating that positive affect is associated with lower levels of PIC), thus promoting faster recovery, and leading to overall better health outcomes.^75^

## Shame as an affective determinant of health?

What we hope to have demonstrated at this point is that our first contention—that shame, both directly and indirectly, impacts on health through a variety of plausible mechanisms—is supported by a reasonable body of research. What about our second, more tentative and preliminary, proposal—that shame could be considered an affective determinant of health?

An ever-expanding literature indicates that population and individual-level health outcomes are attributable to a wide variety of factors such as social connectedness, social capital, gender, urban living environment, race and many others. Perhaps the most widely investigated of these over the past 50 years has been the relationship between social status and health.^76^ ^77^ In general, the studies concur—social disadvantage is correlated with worse health.^78^ Because poorer people tend to have poorer health (although this is not an inevitable association), poverty is generally classed as a social determinant of health.^42^ Despite the abundance of research, some complex questions remain unelucidated, including the mechanisms through which factors such as poverty impact on health.^78^ Macleod *et al* 78 suggest that the debate over the origin of health inequalities has ‘polarised around two basic explanatory hypotheses. These have been referred to as the “material” and the “psychosocial” explanations of health inequalities, respectively’. The psychosocial hypothesis posits it is the ‘psychological stress’ associated with perceptions of disadvantage that is health damaging.^78^ While this debate is far from settled, the question might be asked—what is there to gain by presenting shame as an affective determinant of health, rather than simply subsuming it into the broader psychosocial hypothesis that attempts to explain the relationship between poverty, or race, or gender, or other minority stresses, and health outcomes? We suggest that there would seem a number of advantages in advancing an affective hypothesis that isolates shame as an affective determinant of health.

First, as Greco and Stenner state, ‘concern with emotion…has come to be a shared focal point for an emerging community of scholars’.^6^ This is not simply an intellectual ‘affective turn’ within the literature of disciplines, but rather is responsive to, and revealing of, the increasing importance of ‘the emotional’ in many facets of interaction and communication in postmodern societies.^6^ The incorporation of emotion into theories about health and illness is thus not simply the imposition of something novel on to an existing theoretical framework, but instead has the potential to reflect ‘affective life’ and transform the conceptual landscape.^79^ ^80^ Engagement with affect opens up the possibility of regarding shame as a scientific category, an object of health research linked to the political dimensions of governance, morality and the normative business of the regulation of bodies. Shame, as we have seen, is common to many negative social processes, mediating relations between power and persons and contributing to affecting physical and psychological experiences. Chronic shame is hidden within populations and minority stress identities. Critical reflection on how it originates, persists and pervades the dynamics of contemporary social, and self, governance and contributes to forms of affective consciousness that are problematic and unhealthy, opens up the possibility of enhancing visibility and sensitivity, and potentially alleviating negative impacts.

Second, shame is a ubiquitous emotion in the clinical environment because of the potential for exposure of perceived defects, inadequacies or illness. Shame may arise inadvertently in the consultation, or indeed be brought about deliberately by the clinician. In a world where a significant proportion of ill-health (and mortality) is directly or indirectly attributable to health-related behaviours such as smoking, diet, exercise, alcohol and substance abuse, there has been a drive to tackle these issues through direct confrontation, or through more subtle ‘nudging’. While there seems little consensus on which approach might be most effective in bringing about the medically desired behavioural change, directly confronting, admonishing, or perhaps even ‘nudging’, patients runs the risk of eliciting the response of shame to observed criticism. This may have significant consequences for both the patient's health and the doctor-patient relationship. It is undoubted that shame may have a positive impact on the health of some individuals through promoting healthy behaviour but, as Harris and Darby^34^ point out ‘although one can hardly fail to be impressed by the proportion of people attributing benefits to shame-provoking interactions, the proportions of individuals who ceased contact with or lied to a physician might reasonably be judged unacceptably high’. This leaves us in the difficult position of not knowing who shame will affect, and how, and whether the overall outcome for the individual or the population will be positive or negative on a utilitarian calculus. Shaming an individual, or deploying a shaming campaign across a population, offers no guarantee that the targeted subject or subjects will respond in a particular way. There is of course the question of whether this is the right moral framework to apply in the first instance. Regardless of this, bringing shame to the centre of the medical paradigm will ensure that health professionals are attuned to the dynamics of shame within clinical contexts, where shame on the part of patients can lead to avoidance and concealment.

Third, the link between shame and health is not simply about a human propensity for embarrassment regarding the physical body and a related concern regarding judgement from healthcare professionals, but there is a significant political and socioeconomic dimension. When individuals are stigmatised or marginalised for reasons that may well have nothing to do with their health status, such as for poverty, race or gender, they may well experience shame as a chronic state of their subjectivity as a result of persistent experiences of social evaluative threats. These chronic feelings of global personal inadequacy may well have a substantially negative effect on health behaviours and health outcomes. Thus, it is pertinent that doctors become accustomed to considerations of affective responses but, as pointed out by Viney *et al*,^81^ there are many ‘other kinds of relations, networks, nodes and entities through which health and medicine are made, and unmade’ that must be included in any conceptualisation or analysis of health and illness. Designating shame as an affective determinant of health would raise awareness of the links between the cultural politics of shame—namely, the dynamics of inclusion and exclusion, social-evaluative threats and status anxiety—and negative health outcomes. Beyond this, the contemporary tendency towards a politics of personal responsibility in clinical care, where the idea is that everyone is capable of modifying and controlling their behaviour and lifestyle and, hence, responsible for their own risk factors, through their capacity to make ‘wise choices’, could be re-evaluated. It is clear that socioeconomic inequalities mean that our capacities for the sorts of choices we make about our health behaviours can be drastically unequal, and that this inequality in itself can have a concrete effect on health, despite the positive or negative material conditions in which we find ourselves. As a result, the use of stigma and shame in public health campaigns and as a strategy to motivate for healthy behaviours, for example, when considering conditions such as obesity, sexual health and addiction, where individuals are seen to be making ‘choices’ that affect their health status, should be carefully reconsidered. Exacerbating shame for populations that are likely to be dealing with chronic shame and marginalisation may lead to a worsening, rather than an attenuation, of overall negative health outcomes.

## Conclusion

Within this paper, we have outlined how shame may impact on the health of individuals and populations. We believe that its influence is sufficiently powerful for it to be considered an important contributor, or even determinant, of health status. This is in no way to propose that it acts independently of other material or psychosocial factors, or that it is mediated differently within the body than other stress responses. Rather, our claim is that shame is so insidious, pervasive and pernicious, and so critical to clinical and political discourse around health, that it is imperative that its vital role in health, health-related behaviours and illness be recognised and assimilated into medical, social and political consciousness and practice.

However, there are many questions that yet remain unanswered, and so we will conclude with a proposal for a research agenda that aims to extend the state of knowledge, focusing on clarifying the nature of the burden, the populations affected and on the development and assessment of potential strategies aimed at mitigating problematic shame. The research agenda would address the following six broad aims:

1. Identify the determinants and indicators of shame and their practical implications for health policy.
2. Build on the limited research that outlines the responses of individuals to acute shame, identifying perceptual, disease specific, medical, cultural, institutional and sociopolitical factors that mediate reactions and outcomes.
3. Quantitatively and qualitatively assess the burden from stigma and acute shame associated with various health problems, both in terms of nature and magnitude.
4. Explore shame associated with different health problems and in different settings. It is important to determine whether and how shame-proneness for the same issue varies across the multiple sites identified by Viney *et al*.^81^
5. Analyse the role of chronic shame in a variety of health and social environments, and its relationship with suffering and illness in response to acute episodes.
6. Improve knowledge about the functional impact of shaming, and the risk of furthering health problems through health policy and legislation that inadvertently or consciously promotes stigma and shame. Social policy needs to be informed by research, rather than being influenced by stereotype and prejudice.

Shame is an important subject for public health, and the health of the public. It merits serious assessment and discussion. It contributes to suffering, impairs health, interferes with health relationships and impacts on service provision. Emotion is important in social life, and while the social sciences and other disciplines have taken an ‘affective turn’, shame remains the elephant in the clinical room.
